# Privacy Protection Optimization Method for Cloud Platforms Based on Federated Learning and Homomorphic Encryption

**DOI:** 10.3390/s26030890

**Published:** 2026-01-29

**Authors:** Jing Wang, Yun Wang

**Affiliations:** School of Computer Science and Engineering, Southeast University, Nanjing 211189, China; 230238567@seu.edu.cn

**Keywords:** cloud computing, federal study, homomorphic encryption, privacy protection, distributed training

## Abstract

With the wide application of cloud computing in multi-tenant, heterogeneous nodes and high-concurrency environments, model parameters frequently interact during distributed training, which easily leads to privacy leakage, communication redundancy, and decreased aggregation efficiency. To realize the collaborative optimization of privacy protection and computing performance, this study proposes the Heterogeneous Federated Homomorphic Encryption Cloud (HFHE-Cloud) model, which integrates federated learning (FL) and homomorphic encryption and constructs a secure and efficient collaborative learning framework for cloud platforms. Under the condition of not exposing the original data, the model effectively reduces the performance bottleneck caused by encryption calculation and communication delay through hierarchical key mapping and dynamic scheduling mechanism of heterogeneous nodes. The experimental results show that HFHE-Cloud is significantly superior to Federated Averaging (FedAvg), Federated Proximal (FedProx), Federated Personalization (FedPer) and Federated Normalized Averaging (FedNova) in comprehensive performance, Homomorphically Encrypted Federated Averaging (HE-FedAvg) and other five baseline models. In the dimension of privacy protection, the global accuracy is up to 94.25%, and the Loss is stable within 0.09. In terms of computing performance, the encryption and decryption time is shortened by about one third, and the encryption overhead is controlled at 13%. In terms of distributed training efficiency, the number of communication rounds is reduced by about one fifth, and the node participation rate is stable at over 90%. The results verify the model’s ability to achieve high security and high scalability in multi-tenant environment. This study aims to provide cloud service providers and enterprise data holders with a technical solution of high-intensity privacy protection and efficient collaborative training that can be deployed in real cloud platforms.

## 1. Introduction

Inter-organizational data collaboration has become an important foundation of intelligent service and resource optimization. However, the multi-tenant architecture and heterogeneous node characteristics of cloud platforms make data security and privacy protection face severe challenges [1]. The traditional centralized training method requires the aggregation of original data, which not only increases the risk of data leakage, but also is difficult to meet the requirements of data compliance and safe calculation [2]. Federated learning (FL) realizes collaborative modeling by completing training locally and only sharing model parameters, which provides a new technical path for privacy protection and data utilization [3,4]. However, the existing FL still has some problems such as high communication load, low aggregation efficiency and significant influence of heterogeneous nodes in multi-tenant scenarios, which makes it difficult to balance security and performance.

To further improve the privacy computing capability on cloud platforms, a researcher proposed a secure FL scheme combining homomorphic encryption (HE) mechanism to realize parameter aggregation and model update in the ciphertext state. HE protects data confidentiality while avoiding intermediate exposure, but it also introduces significant calculation and communication overhead. Especially in the cloud platform environment with high concurrency and heterogeneous nodes, how to maintain the scalability and training stability of the system under security constraints has become a key issue affecting the practicality of the model [5,6].

Based on this, this study puts forward the Heterogeneous Federated Homomorphic Encryption Cloud (HFHE-Cloud) model, which deeply integrates multi-layer HE structures with heterogeneous adaptive aggregation mechanisms and constructs an optimized privacy protection framework for multi-tenant cloud platforms. In HFHE-Cloud, federated learning is adopted as a distributed model training paradigm. It organizes multi-tenant cloud nodes to complete collaborative modeling without sharing raw data, thereby addressing the problems of data decentralization, non-centralization, and cross-tenant collaborative training in cloud environments. Homomorphic encryption is used to protect the privacy and security of model parameters and gradient updates during the federated learning process, preventing information leakage during parameter exchange and aggregation stages. This study focuses on distributed supervised learning tasks in multi-tenant cloud environments, implementing the model training process through federated learning and ensuring parameter privacy and system security during training via homomorphic encryption. In HFHE-Cloud, the two techniques assume distinct functional roles, with federated learning serving as the training mechanism and homomorphic encryption acting as the secure computing approach. The model realizes efficient ciphertext aggregation through dynamic weight distribution and hierarchical key management, considering data security and distributed performance, and can realize secure and verifiable collaborative training in high-concurrency environments. The innovation of this study is mainly reflected in the following three aspects:

(1) An adaptive aggregation algorithm for heterogeneous nodes is proposed, which dynamically adjusts the participation degree based on the computing power, delay, and failure rate of nodes to achieve the optimal balance and rapid convergence of federated aggregation;

(2) We construct a hierarchical and switchable homomorphic encryption mechanism, the Flexible Homomorphic Encryption Scheme (FHES), to reduce the cost of encryption and decryption and improve the efficiency of ciphertext calculation under the premise of ensuring the security level;

(3) We design a chain audit and dynamic trust evaluation mechanism to realize the traceability and verifiability of cross-tenant collaboration process and enhance the engineering feasibility and system robustness of cloud platform privacy computing.

This study is organized as follows: Section 1 introduces the research background and research motivation, as well as the main research content and contributions of this study. Section 2 reviews and analyzes the research progress related to cloud platform privacy protection, homomorphic encryption, and federated learning, clarifying the limitations of existing methods. Section 3 elaborates on the overall architecture, key mechanisms, and implementation principles of the HFHE-Cloud model, including the federated learning process, homomorphic encryption, and key management strategies. Section 4 presents the experimental design and performance evaluation results, verifying and analyzing the proposed method in terms of accuracy, latency, system overhead, and scalability. Finally, Section 5 summarizes the work of the full paper and discusses potential future research directions.

## 2. Related Work

### 2.1. Research Progress of Privacy Protection and Encryption Technology on Cloud Platforms

The development of cloud computing has significantly improved the convenience of data storage and sharing, but at the same time it has also brought severe challenges to data privacy and security. Chowdhury et al. pointed out that with the rapid increase in the demand for image storage in the cloud, although traditional encryption methods can prevent unauthorized access, they often ignore the usability of encrypted images. Therefore, they put forward the Thumbnail Preservation Encryption (TPE) method, which can still preview an image after encryption and improve the encryption speed by about 17 times while maintaining privacy. They showed that the Structural Similarity Index Measure (SSIM) value was close to 0.9 and the Number of Pixels Change Rate (NPCR) value reached 99%, effectively balancing usability and security [7]. Dewangan et al. discussed the vulnerability of cloud storage systems in the face of Chosen Ciphertext Attack (CCA) from the perspective of cryptography and emphasized that public key encryption systems should have adaptive anti-attack ability [8]. Qiu et al. further proposed the Privacy-Preserving Traceable Functional Encryption for Inner Product (PPT Fe-IP) scheme, which realizes the balance between identity privacy and traceability through a two-party secure computing protocol between a Key Generation Center (KGC) and users [9]. Hu et al. paid attention to the hidden danger of Searchable Encryption (SE) in the threatening attack scenario and pointed out that the existing scheme was not secure enough when users were forced to disclose search records [10]. Nataraj and Nataraj put forward an optimization mechanism to improve the processing efficiency and reduce the risk of data leakage in view of the privacy and delay of cloud object storage systems (COSSs) in multi-party data sharing [11].

### 2.2. Privacy Protection Method Based on FL and Homomorphic Encryption

In recent years, FL has attracted much attention because it can realize collaborative modeling without concentrating the original data. Yang et al. combined blockchain, HE, and the reputation mechanism and proposed an encrypted FL framework based on blockchain, which used the reputation incentive mechanism to enhance the contribution of participating nodes and ensure the privacy and security of model training [12]. Mahato et al. proposed Privacy-Preserving Verifiable Federated Learning (PPV FL) to further integrate blockchain and HE and used the Elliptic Curve Digital Signature Algorithm (ECDSA) and Byzantine Fault Tolerance (BFT) to strengthen system security [13]. Kumbhar and Rao designed a multi-key HE model through the Puzzle Archimedes Optimization Algorithm (PAOA) to realize the secure sharing of distributed medical data, and its training accuracy on the Cleveland dataset reached 0.946 [14]. Anitha and Murugan proposed Modified Homomorphic Encryption Federated Adaptive Hybrid Dandelion Search (MHEF-AHDS), which combined the blockchain Internet of Things (IoT) and quantum machine learning technology to optimize the system performance from multiple dimensions such as throughput, energy consumption, and F1 score [15]. ChandraUmakantham et al. introduced the enhanced Ghost Bidirectional Network (Ghost_Binet) model and improved the accuracy of an intrusion detection system (IDS) through Chaotic Chebyshev Artificial Hummingbird (CaH) and HE [16]. Castro et al. aimed at the problem of the high communication and calculation cost of existing privacy-preserving federated learning based on homomorphic encryption (PPFL-HE). They proposed the Dynamic Range Evaluation Layer-Wise (DREL) quantization strategy. The Brakerski/Fan-Vercauteren (BFV) algorithm was adopted, which reduced the encryption time by about 99% compared with the traditional scheme [17]. Park et al. proposed a secure aggregation method combining masking aggregation and multi-key HE and realized model parameter aggregation without node interaction by using the Cheon–Kim–Kim–Song (CKKS) scheme, which significantly reduced the number of communication rounds [18]. Saidi et al. used Cipher Text-Policy Attribute-Based Encryption (CP-ABE) to perform homomorphic encryption with CKKS to construct an efficient and secure model aggregation mechanism, which improved the balance between privacy protection and performance [19].

In addition, to address practical issues prevalent in multi-tenant cloud environments, including non-independent and identically distributed (non-IID) data, limited communication, asynchronous updates, and the involvement of malicious nodes, Miao et al. proposed the Motivated Contrastive Federated Learning with Compressive Sensing (MCFL-CS) method. This method alleviated the impact of data heterogeneity on federated training accuracy by fusing contrastive loss and cross-entropy loss on the client side. It also combined an improved compressive sensing mechanism and local differential privacy technology, significantly reducing communication costs while satisfying formal privacy protection requirements. Experimental results showed that the MCFL-CS method improved the average model accuracy by 3.45% under the non-IID setting across multiple datasets and reduced communication overhead by more than 95% [20]. Furthermore, targeting the accuracy degradation caused by model update delays in asynchronous federated learning, Miao et al. proposed the Privacy-preserving Asynchronous Federated Learning (PAFed) framework based on the Alternating Direction Method. This framework corrected inconsistencies between delayed updates and current global updates via vector projection technology. They also introduced an optimization strategy based on the Alternating Direction Method of Multipliers (ADMM) to enhance convergence performance under heterogeneous data distribution conditions. Experimental results demonstrated that the PAFed framework achieved a maximum accuracy improvement of 12.53% compared with Federated Learning based on the Alternating Direction Method of Multipliers (FedADMM) [21]. Meanwhile, considering the risk of model poisoning attacks faced by federated learning systems in multi-tenant cloud environments, Miao et al. further proposed the Robust Federated Learning (RFed) framework with scaled dot-product attention under dual-server architecture. By introducing a dual-server architecture and scaled dot-product attention mechanism, this framework significantly enhanced the robustness and prediction accuracy of the federated training process without relying on strong assumptions. It also effectively reduced computational overhead while maintaining privacy protection capabilities, resulting in a failure rate of poisoning attacks exceeding 96% [22]. Overall, these methods enhance the applicability of privacy-preserving federated learning in complex cloud environments from the perspectives of communication efficiency optimization, asynchronous aggregation stability, and malicious behavior defense, respectively. However, they still fail to systematically solve the collaborative optimization problem of heterogeneous node scheduling, key management, and homomorphic encryption computing efficiency in multi-tenant cloud platforms.

### 2.3. Existing Research and Analysis

The existing research has made much progress in the direction of privacy protection by combining FL with homomorphic encryption, and the proposed schemes are outstanding in security and accuracy. However, most of these studies focus on specific fields or small-scale environments, and there is a lack of targeted adaptation to the complex structure of cloud platforms. For instance, the multi-key homomorphic encryption federated learning proposed by Kumbhar and Rao was mainly designed for distributed medical data sharing scenarios. Its experimental verification relied on specific medical datasets, making it difficult to reflect the characteristics of high concurrency and heterogeneous resource scheduling on multi-tenant cloud platforms [14]. Anitha and Murugan [15] and ChandraUmakantham et al. [16] focused on blockchain-based Internet of Things environments and intrusion detection systems, respectively. Their model designs were highly dependent on application scenarios, lacking adaptability analysis for general cloud computing environments. Meanwhile, the studies conducted by Castro et al. [17], Park et al. [18], and Saidi et al. [19] focused on the optimization of secure aggregation protocols or homomorphic encryption computing efficiency. They placed more emphasis on cryptography-level security and performance, while insufficiently considering system-level issues such as multi-tenant collaboration, node heterogeneity, and dynamic resource scheduling on cloud platforms.

Cloud platform data is characterized by multi-tenant concurrency, heterogeneous nodes, and dynamic resource scheduling, which leads to uneven communication delay and difficult synchronization [23,24]. The data privacy of a cloud platform lies in not the logical isolation among tenants but the sharing of resources. Once the aggregation protection is insufficient, it is prone to parameter leakage or reasoning attacks [25]. Although FL can avoid the risks brought by data concentration, it still faces the trade-off between convergence efficiency and security under the conditions of high concurrency and dynamic nodes. Most of the research is based on a static key system and fixed aggregation strategy, and the dynamic trust of tenants and key mapping management are not fully considered. With the rise in data-centric computing services, the lack of dynamic encryption aggregation mechanisms for multi-tenant heterogeneous scenarios has become a key bottleneck restricting the deployment of cloud-based FL. Facing the requirements of multi-source sharing and real-time secure computing, it is necessary to change the traditional design ideas of single-layer encryption and centralized aggregation. Although the existing research considers the integration of FL and homomorphic encryption, it lacks the comprehensive optimization of the dynamics and scalability of cloud environments. Therefore, this study proposes a hierarchical homomorphic aggregation and trust adaptive mechanism through HFHE-Cloud. It optimizes performance through multi-layer key mapping and asynchronous parameter scheduling, while ensuring privacy integrity and system credibility in multi-tenant collaboration on cloud platforms.

## 3. Realization Principle and Analysis Process of HFHE-Cloud Model

### 3.1. Realization Principle of HFHE-Cloud Model

The HFHE-Cloud model is based on the deep integration of FL and HE and is oriented to multi-tenant, heterogeneous nodes and the highly concurrent-data environment of cloud platforms to achieve high security and high scalability in cross-tenant collaborative learning. The whole model consists of four logical layers: the data access and local computing layer, encryption and key management layer, aggregation and collaborative optimization layer, and global control and audit layer. The four layers are connected by an encryption channel, a key mapping and dynamic trust evaluation mechanism, which realizes the full-cycle privacy closed loop from data generation to model update.

#### 3.1.1. Data Access and Local Computing Layer

The data access and local computing layer undertakes the basic task of model training. Multiple tenant nodes on the cloud platform perform local model training according to resource conditions and task division, and each node independently processes its own data to avoid the leakage of original data. In this layer, heterogeneous adaptive aggregation algorithm and tenant-isolation multi-task FL algorithm are introduced. The former dynamically adjusts the training step size and parameter upload period according to multi-dimensional indicators such as node computing power, communication delay, and the failure rate. Specifically, the effective participation degree $\rho_{k}^{\left(t\right)}$ of node k in round t of training is calculated by the following Equation (1):

(1) $$\rho_{k}^{\left(t\right)}=\frac{\alpha \cdot {\mathrm{CompPower}}_{k}}{\beta \cdot {\mathrm{Latency}}_{k}^{\left(t\right)}+\gamma \cdot {\mathrm{FailureRate}}_{k}}\cdot exp(-\lambda \cdot t)$$

α,β,γ and λ are balance coefficients to realize load balancing and delay compensation among heterogeneous nodes. The latter divides the model into public feature layer and tenant-specific layer through the hierarchical sharing design of the model structure. Let the global shared parameter be $w_{g}$ and the exclusive parameter of tenant k be $w_{k}^{s}W$ and its local loss function $F_{k}$ can be expressed as Equation (2):

(2) $$F_{k}(w)=F_{k}(w_{g},w_{k}^{s})=E_{(x,y)∼D_{k}}[\ell (f(x;w_{g},w_{k}^{s}),y)]+\lambda_{1}R_{1}(w_{g})+\lambda_{2}R_{2}(w_{k}^{s})$$

The function f(·) denotes a feedforward neural network composed of the multi-layer fully connected structure of a Multilayer Perceptron (MLP). It is used to characterize the nonlinear mapping relationship between input features and prediction targets, and its parameters consist of shared parameters $w_{g}$ and tenant-specific parameters $w_{k}^{s}$. ℓ is the loss function, and $R_{1}$ and $R_{2}$ are regularization terms acting on shared parameters and exclusive parameters. The local model solves the optimal parameters by minimizing the loss function (see Equation (3)):

(3) $$w_{k}^{\left(t\right)}\;=\;\arg\;\underset{w}{\min}\;F_{k}(w;D_{k})$$

#### 3.1.2. Encryption and Key Management Layer

The encryption and key management layer constitutes the core security support of the system. It should be noted that the only encrypted objects in HFHE-Cloud are the model parameter update vectors uploaded by each tenant node during the federated learning training process. The original tenant data is always stored in local computing nodes and does not participate in any form of encrypted transmission or cloud-side processing. This layer adopts a hierarchical selective encryption and segmented Flexible Homomorphic Encryption Scheme (FHES) mechanism. This study does not propose new homomorphic encryption primitives; the adopted FHES is an engineered combination mechanism based on existing lattice-based homomorphic encryption frameworks. Its underlying security relies on the well-established Ring Learning with Errors (RLWE) assumption. Based on addition, multiplication, and key switching operations supported by existing homomorphic encryption algorithms, it introduces hierarchical selective encryption and multi-key mapping strategies to adapt to federated learning and multi-tenant cloud environments. For the highly sensitive parameter vector $\;\vec{m}\in R_{q}^{n}$ (where $R_{q}$ is a polynomial ring of module q), the encryption process can be expressed as Equation (4):

(4) $$\vec{c}=\mathrm{FHES}.\mathrm{Enc}(\vec{m},pk)=[\vec{a}\cdot \vec{s}+\vec{e}+\Delta \cdot \vec{m}{]}_{q}$$

$pk=(\vec{a},\vec{b})$ is the public key, $\vec{s}$ is the private key, $\vec{e}$ is the small error term, and Δ is the scaling factor. This scheme ensures that the ciphertext maintains linear combinability in the operation stage. FHES serves as the homomorphic computing support mechanism in this study. Its functional scope is limited to supporting homomorphic addition, homomorphic multiplication, and key switching operations across key spaces for model parameter updates, without involving structural modifications to key generation algorithms or underlying encryption primitives. The system-level innovations are mainly reflected in the parameter sensitivity-driven encryption strategy, key mapping scheduling, and collaborative design with the federated learning process, rather than modifications to the mathematical structure of homomorphic encryption itself.

To quantitatively describe the privacy sensitivity of model parameters, the system calculates the parameter sensitivity $S(m_{i})$ of the parameter vector $\vec{m}\in R_{q}^{n}$ in the local training stage, which is defined as the average gradient amplitude of the parameter to the local loss function:

(5) $$S(m_{i})=E_{(x,y)∼D_{k}}\left[|\frac{\partial L(x,y;\theta )}{\partial m_{i}}|\right]$$

L(·) denotes the local loss function, and $D_{k}$ represents the local data distribution of node k. Parameter sensitivity reflects the degree to which changes in a single parameter affect the model’s decision outcomes. The system classifies parameters into hierarchical categories based on a preset threshold τ. When $S(m_{i})\geq \tau$, the parameter is identified as a highly sensitive parameter and protected by homomorphic encryption. Otherwise, lightweight encryption or plaintext uploading strategies are adopted. This sensitivity-driven selective encryption mechanism ensures privacy and security while effectively avoiding over-encryption of low-impact parameters, thereby reducing the overall encryption and decryption overhead and improving system efficiency.

FHES algorithm adopts adaptive multi-modulus operation strategy in ciphertext space, and homomorphic addition of two ciphertexts, ${\vec{c}}_{1}\;\mathrm{and}\;{\vec{c}}_{2}$, is directly performed in ciphertext space (Equation (6)):

(6) $${\vec{c}}_{\mathrm{sum}}=[{\vec{c}}_{1}+{\vec{c}}_{2}{]}_{q}$$

After decryption, it satisfies $\left[{\vec{m}}_{1}+{\vec{m}}_{2}]\approx \mathrm{FHES}.\mathrm{Dec}({\vec{c}}_{\mathrm{sum}},sk\right)$. For homomorphic multiplication, the system manages noise growth by dynamically expanding polynomial ring domain and analog-to-digital conversion (see Equation (7)):

(7) $${\vec{c}}_{\mathrm{prod}}\approx \mathrm{FHES}.\mathrm{Mult}({\vec{c}}_{1},{\vec{c}}_{2})\Rightarrow \mathrm{FHES}.\mathrm{Dec}({\vec{c}}_{\mathrm{prod}},sk)\approx [{\vec{m}}_{1}⊙{\vec{m}}_{2}]$$

⊙ means multiplication by elements. Each node has an independent public–private key pair $\left(pk_{k},sk_{k}\right)$, and the security module generates a global public key PK for aggregation. The key conversion process allows ciphertext encrypted with different public keys to be converted into a format that can be processed with the same aggregate key. For the ciphertext ${\vec{c}}_{k}=\mathrm{Enc}(pk_{k},{\vec{m}}_{k})$ of node k, the process of transforming it into ciphertext ${\vec{c}}_{k}^{\prime }$ under the global public key PK can be modeled as Equation (8):

(8) $${\vec{c}}_{k}^{\prime }=\mathrm{KeySwitch}({\vec{c}}_{k},\tau_{sk_{k}\to SK})$$

$\tau_{sk_{k}\to SK}$ is the conversion key from the node private key $sk_{k}$ to the system aggregation key SK. In specific implementation, the switching key $\tau_{sk_{k}\to SK}$ is locally generated by node k. Its construction process is based on the node private key $sk_{k}$ and the aggregation key SK issued by the system, which is used to support the secure conversion of ciphertext representations across different key spaces. The KeySwitch operation first uses $\tau_{sk_{k}\to SK}$ to expand and relinearize the private key-related components in the ciphertext ${\vec{c}}_{k}$. Then, it completes linear combination and noise control in the ciphertext domain and re-represents it as the equivalent ciphertext ${\vec{c}}_{k}^{\prime }$ under the aggregation key SK. The entire key switching process is performed in the ciphertext space, without requiring the server or other nodes to access the node private key. Under this encryption and aggregation process, the server only touches the homomorphically encrypted model parameter updates and cannot directly obtain the plaintext parameters or intermediate training results of any tenant node. The hierarchical selective encryption mechanism further limits the scope of information exposure of highly sensitive parameters during aggregation. Meanwhile, the multi-key mapping and key switching operations ensure the computability of ciphertexts from different nodes in a unified aggregation key space. Together, these mechanisms reduce the risk of model parameters being inferred or reconstructed during cross-node collaboration and cloud-side aggregation. They enable the system to maintain consistent privacy isolation at the parameter level, thereby ensuring the security and consistency of cross-node homomorphic aggregation.

#### 3.1.3. Aggregation and Collaborative Optimization Layer

The aggregation and collaborative optimization layer are responsible for the encryption aggregation and dynamic optimization of the global model. The server performs homomorphism operation on ciphertext updates from different tenants without decryption. Suppose that the encryption parameters of each node are updated to $\{{\vec{c}}_{k}^{\left(t\right)}{\}}_{k=1}^{K}$ in the t round of training. Global aggregation operations are performed under homomorphic conditions (Equation (9)):

(9) $${\vec{c}}_{\mathrm{global}}^{\left(t\right)}=\sum_{k=1}^{K}\eta_{k}^{\left(t\right)}\cdot {\vec{c}}_{k}^{\left(t\right)}$$

$\eta_{k}^{\left(t\right)}$ is the dynamic weight assigned to node k. The built-in weighted aggregation control module in this layer dynamically allocates the weight according to the node contribution, training accuracy and communication frequency. A feasible way to calculate the weight is to combine the local data quantity $n_{k}$ of a node with its model updating quality $q_{k}^{\left(t\right)}$ (such as the decline rate of local loss after the last round of aggregation) (see Equation (10)):

(10) $$\eta_{k}^{\left(t\right)}=\frac{n_{k}\cdot exp(q_{k}^{\left(t\right)}/T)}{\sum_{j=1}^{K}n_{j}\cdot exp(q_{j}^{\left(t\right)}/T)}$$

T is a temperature parameter used to control the smoothness of the distribution. To ensure the correctness of the aggregation results, the server will generate a verifiable proof $\Pi_{\mathrm{agg}}$, and its verification process can be expressed as Equation (11):

(11) $$\mathrm{Verify}(\Pi_{\mathrm{agg}},\{{\vec{c}}_{k}^{\left(t\right)}\},{\vec{c}}_{\mathrm{global}}^{\left(t\right)})≟1$$

#### 3.1.4. Global Control and Audit Layer

The global control and audit layer constitutes the core of the system’s trust guarantee. This layer records the key operations and node interaction behavior of each round of training through chain audit log and zero knowledge verification mechanism. The system can check the calculation compliance of nodes without exposing the model content. For example, the auditor can ask the node to provide zero-knowledge proof $\pi_{k}$ of the effectiveness of its local training process, which proves that it has indeed performed the specified training steps on the local dataset $D_{k}$ without revealing $D_{k}$ or intermediate parameters. The generating relation of the proof can be formally defined as Equation (12):

(12) $$\pi_{k}\leftarrow \mathrm{ZK}-\mathrm{Prove}\{\mathrm{Private}:\;D_{k},w_{k}^{\left(t-1\right)};\mathrm{Public}:\;w_{k}^{\left(t\right)},\mathrm{Com}\}:\mathrm{Update}(D_{k},w_{k}^{\left(t-1\right)})=w_{k}^{\left(t\right)}$$

Com is the relevant commitment. In addition, the system maintains a chained audit log L, and every time a new record $\log^{\left(t\right)}$ is added, it is bound to the previous state through a hash function to ensure that it cannot be tampered with (Equation (13)):

(13) $${\mathrm{Hash}}^{\left(t\right)}=H({\mathrm{Hash}}^{\left(t-1\right)}||\log^{\left(t\right)})$$

To evaluate the reliability of nodes, the system continuously calculates the dynamic trust degree $T_{k}^{\left(t\right)}$ of each node, which is updated based on the consistency of its historical behavior $h_{k}$ and the pass rate of audit certification $r_{k}$ (see Equation (14)):

(14) $$T_{k}^{\left(t\right)}=\sigma \cdot T_{k}^{\left(t-1\right)}+(1-\sigma )\cdot tanh(\phi_{h}\cdot h_{k}+\phi_{r}\cdot r_{k})$$

σ is the forgetting factor. $\phi_{h}\;\mathrm{and}\;\phi_{r}$ are the weight coefficients. Finally, through the cooperation of the above multi-layer mechanisms, the system realizes the safe and efficient global model update in the ciphertext state, and the global model $W^{\left(t+1\right)}$ of the t+1st round is generated by the following Equation (15):

(15) $$W^{\left(t+1\right)}=\mathrm{FHES}.\mathrm{Dec}({\vec{c}}_{\mathrm{global}}^{\left(t\right)},SK)+\xi \cdot \nabla R(W^{\left(t\right)})$$

ξ is the gradient weight of regularization term, and R is the regularization function of global model. The hierarchical model realizes the structural integration among secure computing, resource scheduling, and privacy protection and lays the technical foundation for high-intensity privacy protection and distributed intelligent learning in the cloud platform environment.

During the entire operation of HFHE-Cloud, the four logical layers form a closed-loop collaborative mechanism in the order of “training–encryption–aggregation–control“. The data access and local computing layer is responsible for completing local model training on each tenant node and generating parameter updates. Subsequently, these parameter updates are transmitted to the encryption and key management layer, where hierarchical selective encryption is performed based on parameter sensitivity, and the ciphertext representations under the unified aggregation key space are achieved through the key mapping mechanism. The encrypted parameters are subject to homomorphic aggregation and weight adjustment by the aggregation and collaborative optimization layer without decryption, generating global ciphertext model updates. Finally, the global control and audit layer conducts consistency verification and trust evaluation on the processes of training, aggregation, and key usage and feeds control feedback into the next round of local training, thereby realizing an iterative optimization process with cross-layer linkage. The description of the hierarchical structure and interaction relationships of HFHE-Cloud is shown in Table 1.

### 3.2. Analysis Process of HFHE-Cloud Model

#### 3.2.1. Experimental Data Collection Process

**Figure 1:**
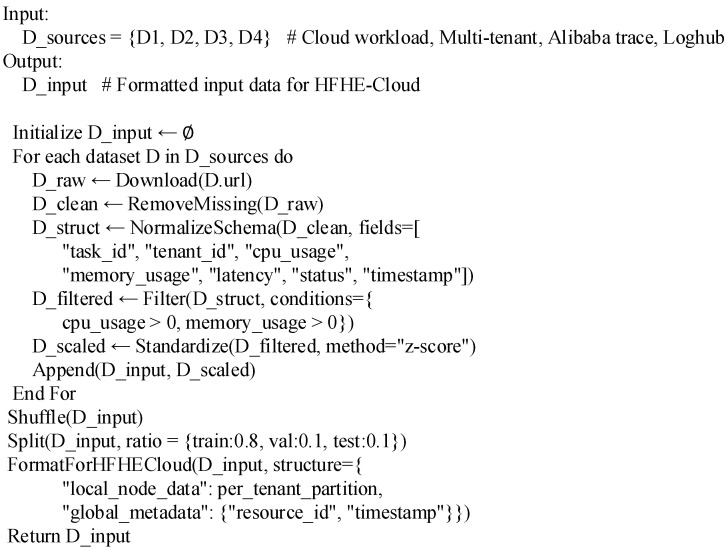
Pseudo-code of data acquisition and input process in HFHE-Cloud platform.

The goal of model design is to build a privacy protection optimization framework with high security and scalability in multi-tenant cloud environment, so the research direction mainly focuses on two dimensions: privacy protection effect and distributed training performance. Considering the distributed, heterogeneous, and highly concurrent characteristics of cloud platform services, the experiment needs to verify the performance of the model under large-scale real task load, and it is difficult for personal collection to cover multi-node and multi-tenant operation scenarios, so this study chooses public authoritative datasets as the experimental basis. The data used are all from well-known research platforms and open resources of enterprises, with complete records, real sources and traceability characteristics, which can accurately reflect the resource allocation, task execution, and security incident behavior in the cloud computing environment. To ensure the representativeness and consistency of the data, all datasets are preliminarily reviewed after collection. After verification, the data content is complete and the structure meets the input standard of HFHE-Cloud model, so they can be directly used as the data source for model training and verification without additional reconstruction or synthesis. The experimental dataset description and pseudo-code of HFHE-Cloud model are shown in Table 2 and Figure 1, respectively.

#### 3.2.2. Experimental Environment

To ensure the repeatability and stability of HFHE-Cloud model under distributed and high security conditions, the experiment is completed in a unified software and hardware environment, and the specific configuration is shown in Table 3.

In terms of specific deployment methods, the experiment implements the HFHE-Cloud framework based on a Kubernetes-enabled containerized distributed environment. All experimental nodes operate within the same physical cluster and are instantiated via Docker containers, where each container corresponds to one federated learning client node and the aggregation server runs as an independent service node. The experiment configures a total of 1 aggregation server node and 20 client nodes. The client nodes are logically divided according to the multi-tenant mode, with each tenant consisting of 1–2 client containers. Tenant-level resource isolation and scheduling management are achieved through Kubernetes namespaces. All nodes share a unified hardware resource pool as shown in Table 2. Heterogeneous computing and concurrent operating environments on cloud platforms are simulated among different nodes through container scheduling and resource quota constraints.

To further clarify the configuration details at the experimental implementation level, Table 4 presents the key parameter settings of homomorphic encryption and the main training hyperparameters of the federated learning model adopted in the experiments of this study.

#### 3.2.3. Threshold Determination of HFHE-Cloud Equilibrium Coefficient

To ensure stable convergence and efficient aggregation of HFHE-Cloud in multi-tenant environment, it is necessary to reasonably determine the threshold of balance coefficient to coordinate the dynamic trade-off among computing resources, communication delay, and the node failure rate. An experiment took 20% of all datasets as training samples and was conducted in a multi-node distributed environment. Each node independently completed local training and uploaded encryption parameters. The SAViT-NeRF mechanism in the model contains three core equilibrium coefficients: α represents the proportion of computing resources, β represents the weight of communication delay, and γ represents the constraint of node failure rate. The attenuation factor λ controls the stability of the global iterative process.

**Figure 2:**
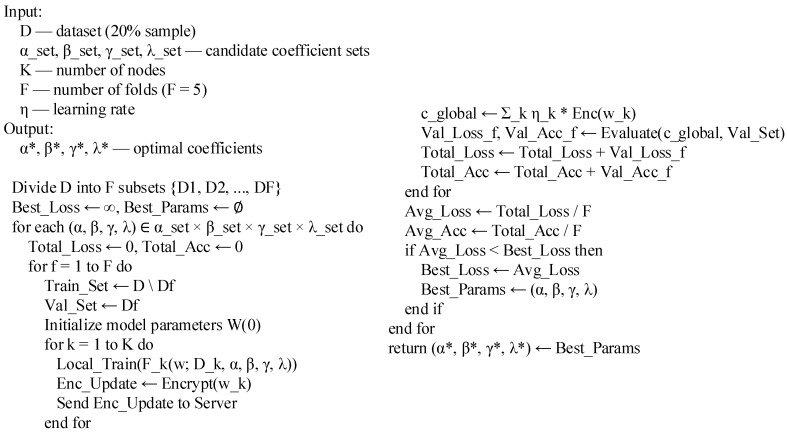
Pseudo-code for determining the threshold of equilibrium coefficient of HFHE-Cloud model.

To obtain the optimal coefficient combination, the method of combining parameter search with 50% cross-validation was studied. The training data was divided into five subsets according to tenants, and four were selected for training and one for verification. The optimal threshold was determined by comparing the verification loss and global accuracy under different parameter combinations. The experiment maintained a uniform learning rate and iteration times to ensure the fairness of the comparison results. Table 5 and Figure 2 show the verification results and implementation pseudo-code, respectively.

Table 5 shows that HFHE-Cloud performs best when α=0.50, β=0.35, γ=0.10, and λ=0.05, with the lowest verification loss and stable accuracy, and was finally used for joint training and performance analysis of subsequent datasets.

#### 3.2.4. Verification Index System of HFHE-Cloud

Based on the collected data, the model was tested considering two dimensions: the privacy protection effect and distributed training performance. The verification index system of HFHE-Cloud is shown in Table 6.

Based on the above indicators and under a unified dataset standard, five baseline models were selected for comparison with the proposed model: ① Federated Averaging (FedAvg); ② Federated Proximal (FedProx); ③ Federated Personalization (FedPer); ④ Federated Normalized Averaging (FedNA); ⑤ Homomorphically Encrypted Federated Averaging (HE-FedAvg) (which introduces HE operations into the standard FedAvg).

During the experiments, client upload latency and communication-related metrics were all obtained through timestamp statistics in the actual operation process. Specifically, in each training round, after completing local parameter updates, each client recorded the start and end times of encrypted parameter uploading, and the difference between the two was used to calculate the upload latency metric. After receiving all client updates and completing homomorphic aggregation, the server recorded the corresponding time, which was applied to count the aggregation time and communication rounds. The size of parameters uploaded per round was determined by the model parameter dimensions and numerical precision, which remained consistent across all experiments to ensure the fairness of comparison between different methods. All latency data were derived from actual measurements in the containerized distributed operating environment, rather than simulation or manual settings.

## 4. Analysis on the Optimization Effect of Privacy Protection of HFHE-Cloud Model Cloud Platform

### 4.1. Analysis of Privacy Protection Effect

**Figure 3:**
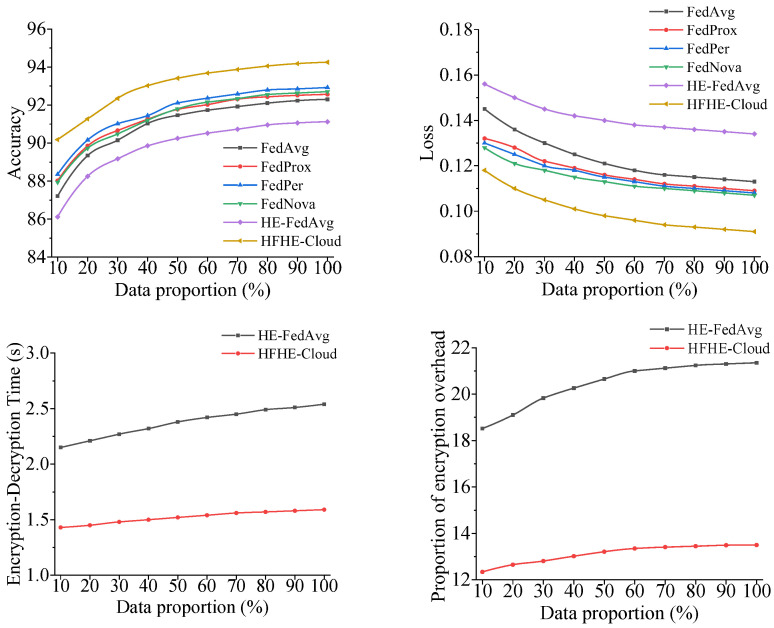
Analysis results of privacy protection effect. Note: Encryption–decryption time and proportion of encryption overhead are only meaningful for the model with encryption mechanism, so only HE-FedAvg and HFHE-Cloud are compared. All the analyses in Figure 3 depend on the hardware environment in Table 3.

The analysis results of privacy protection effect under different data volumes of each model are shown in Figure 3.

According to Figure 3, with the increase in data scale, the accuracy of all models rises, but HFHE-Cloud always maintains the highest accuracy (up to 94.25%) in the whole data range, which is about 2.0–3.0% better than the traditional FedAvg and HE-FedAvg, indicating that it can still maintain a high global convergence accuracy under HE constraints. The loss index further verifies this result. The minimum loss of HFHE-Cloud in the multi-tenant scenario is maintained at about 0.09, and the convergence curve is stable, indicating that the model still has stable gradient optimization performance under the condition of encryption calculation.

In terms of computational efficiency, HFHE-Cloud significantly shortens the encryption and decryption time by introducing hierarchical key mapping and lightweight FHES mechanism, with an average of only 1.5 s, which is about 30% lower than that of HE-FedAvg. Its encryption overhead is controlled at about 13% on average, which is nearly 8 percentage points lower than that of HE-FedAvg, indicating that the model achieves a good balance between security and performance. Overall, HFHE-Cloud ensures privacy security and realizes structural optimization among encryption load, communication cost, and training accuracy, which can provide more efficient and credible privacy computing support in multi-tenant cloud environments.

### 4.2. Performance Analysis of Distributed Training

**Figure 4:**
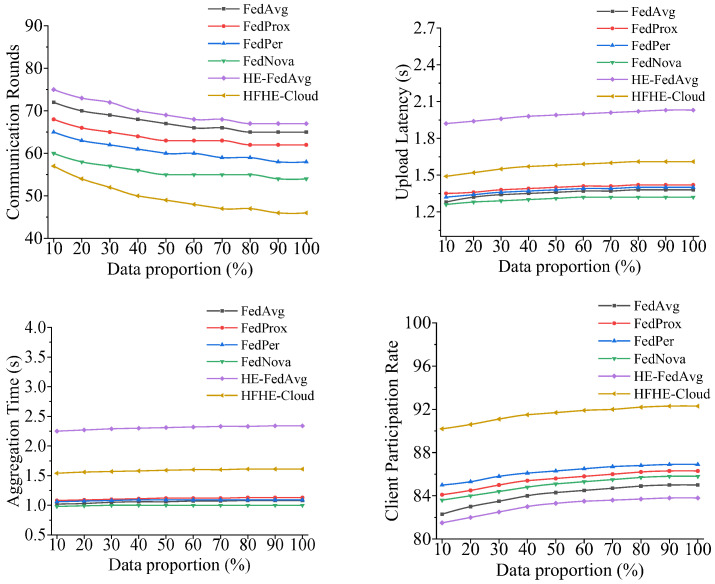
Performance analysis results of distributed training. Note: All the analyses in Figure 4 depend on the hardware environment in Table 3.

The analysis results of distributed training performance of each model under different data volumes are shown in Figure 4.

According to Figure 4, regarding the number of communication rounds, HFHE-Cloud only needs about 46 rounds on average to achieve the same accuracy goal, which is about 20% less than the number needed by FedAvg, which fully reflects the rapid convergence ability of the model under heterogeneous nodes. Its improved dynamic aggregation strategy can effectively restrain the drag of low-performance nodes on the overall training efficiency. Regarding the aspect of upload latency, although HFHE-Cloud includes the HE processes, the average delay is controlled within 1.6 s by layered asynchronous transmission and the parameter compression strategy, which is obviously better than that of HE-FedAvg, which is more than 2.0 s.

The analysis of aggregation performance shows that the aggregation time of HFHE-Cloud under homomorphic encryption is about 1.6 s, which is only slightly higher than that of the unencrypted model, but it is about 30% shorter than that of HE-FedAvg, indicating that its FHES multi-modulus parallel mechanism effectively reduces the computational overhead while maintaining security. In addition, its client participation rate is maintained above 90% for a long time, which is significantly higher than that of the baseline model, reflecting that the system has better node fault tolerance and resource scheduling ability in multi-tenant distributed training. To further evaluate the stability of HFHE-Cloud against the fluctuation of balance coefficients, this study conducted a sensitivity analysis experiment based on the optimal parameter combination given in Table 4 (α=0.50; β=0.35; γ=0.10; λ=0.05). In the experiment, only the communication latency weight β was perturbed and set to 0.9β, β, and 1.1β, respectively, with the remaining parameters kept unchanged. Under the same data scale and number of training rounds, key metrics including encryption and decryption time, the client participation rate, and the number of communication rounds were recorded. The corresponding experimental results are shown in Table 7.

Table 7 shows that when the communication latency weight β is slightly perturbed around the optimal parameter combination, the changes in various performance metrics are generally gentle. When β decreases to 0.9β, the encryption and decryption time decreases slightly, but the number of communication rounds increases and the client participation rate fluctuates mildly. When β increases to 1.1β, both the encryption and decryption time and the number of communication rounds rise, while the client participation rate decreases slightly. In contrast, when β takes the optimal value, it achieves a more stable comprehensive balance among encryption overhead, communication efficiency, and node participation, without obvious deterioration in any single metric. Overall results indicate that HFHE-Cloud has good stability against parameter perturbations near the optimal β, and parameter fluctuations within the range of ±10% will not cause significant changes in system performance. Overall, HFHE-Cloud shows strong advantages in training stability, aggregation efficiency, and system expansibility, which verifies the engineering feasibility and application value of the model in large-scale cloud computing environment.

### 4.3. The Experimental Analysis of Expandable Ablation of HFHE-Cloud

To systematically analyze the scalability sources of HFHE-Cloud under different client scales, this study conducted ablation experiments focusing on the key mechanisms closely related to scale expansion in the model. Specifically, with the dataset, model structure, and optimal balance coefficients shown in Table 4 kept unchanged, the following mechanisms were ablated separately: (1) the heterogeneous perception participation scheduling mechanism (HFHE-Cloud-1); (2) the hierarchical selective encryption strategy (HFHE-Cloud-2); (3) the dynamic weighted homomorphic aggregation mechanism (HFHE-Cloud-3). By gradually removing the above modules and comparing the system performance under different client quantities, each model’s independent contributions to and synergistic effects on system scalability were quantified. The experiment set the number of clients to 20, 40, and 60, respectively, and simulated the scale expansion in multi-tenant cloud environments by adjusting the number of client container instances in Kubernetes. Under each client scale, experiments were performed on both the complete HFHE-Cloud configuration and the corresponding ablation configurations, with key metrics including model accuracy, encryption–decryption time, and communication rounds recorded. The corresponding results are shown in Table 8.

Table 8 shows that across different client scales, the following results are obtained: Removing the heterogeneous perception participation scheduling mechanism (HFHE-Cloud-1) leads to a significant increase in the number of communication rounds, and this effect is gradually amplified as the number of clients rises from 20 to 60. Meanwhile, the client upload latency increases markedly, indicating that in the absence of scheduling constraints, the system is more vulnerable to communication pressure caused by scale expansion. Removing the hierarchical selective encryption strategy (HFHE-Cloud-2) results in higher encryption and decryption time as well as encryption overhead, while the variation in communication rounds is relatively limited. Moreover, such overhead continues to expand with the growth of client quantity, which demonstrates the critical role of selective encryption in controlling computing and resource consumption. Removing the dynamic weighted homomorphic aggregation mechanism (HFHE-Cloud-3) causes simultaneous increases in both communication rounds and latency across all scales, reflecting the importance of aggregation weight adjustment in alleviating large-scale concurrent communication loads. In contrast, the complete HFHE-Cloud configuration maintains relatively stable model accuracy, smoothly increasing latency, and controlled system overhead within the range of 20–60 clients, without obvious performance degradation. This indicates that the system scalability relies on the synergistic effect of the above-mentioned key mechanisms under different scales.

### 4.4. Discussion

From the experimental results, it is shown that the model can complete cross-tenant collaborative training without exposing the original data and maintain high global accuracy and stable convergence performance. This feature is particularly important in the multi-tenant cloud environment: enterprises and institutions often need to share computing power and model power under compliance constraints. While HFHE-Cloud ensures that data is in a safe state during transmission and calculation through encryption aggregation and dynamic trust evaluation mechanisms, which significantly reduces the risk of privacy leakage. At the same time, the layering and adjustability of the FHES effectively control the encryption and decryption overhead. The model avoids the efficiency bottleneck of traditional HE in large-scale training while maintaining high accuracy and verifies the operability of deploying privacy-protected distributed learning in the actual cloud platform architecture.

HFHE-Cloud makes efficient use of computing resources in complex network environments through heterogeneous adaptive aggregation and dynamic scheduling mechanisms of node trust. Its optimization in communication rounds, upload delay, and aggregation time makes the model still have high training stability and scalability under the condition of multi-node concurrency, which is especially critical for realistic cloud computing platforms. Especially in the fields of finance, medical care, e-commerce, etc., data is scattered and security requirements are extremely high. The architecture of HFHE-Cloud can realize joint modeling and intelligent decision-making on the premise of ensuring compliance and privacy.

## 5. Conclusions

The proposed fusion mechanism provides a framework path for cloud privacy computing, but there is still room for improvement. Firstly, the homomorphic encryption and federated aggregation strategy of HFHE-Cloud depends on the preset key hierarchy, which may introduce some key management delay when nodes change dynamically or tenants migrate frequently, thus affecting the real-time performance of the system. Secondly, although the experimental environment covers typical heterogeneous nodes and multi-tenant conditions, it does not fully simulate the influence of cross-regional communication and asynchronous network fluctuation in real cloud platforms. In the future, a more efficient trust evaluation model will be built, and it will be verified in a real cloud platform environment for a long time to further improve the stability and engineering adaptability of HFHE-Cloud in large-scale distributed privacy computing.

## Figures and Tables

**Table 1:** Description of hierarchical structure and interaction of HFHE-Cloud.

Logical Layer	Main Input/Output	How to Interact with Other Layers
Data Access and local computing layer	Input: local tenant data; Output: model parameter update $w_{k}^{\left(t\right)}$.	Submit the locally trained parameter updates to the encryption and key management layer, and receive the scheduling and weight feedback from the control layer.
Encryption and key management layer	Input: model parameter update; Output: encryption parameter ${\vec{c}}_{k}^{\left(t\right)}$.	The parameters from the local computing layer are encrypted in layers and mapped to the aggregation key space through the KeySwitch to provide unified ciphertext for the aggregation layer.
Aggregation and collaborative optimization layer	Input: multi-node ciphertext parameters; Output: global ciphertext model ${\vec{c}}_{\mathrm{global}}^{\left(t\right)}$.	Complete homomorphic aggregation and weight distribution in ciphertext space, and submit the results to the control layer for verification and decryption.
Global control and audit layer	Input: aggregate results and behavior records; Output: control and trust feedback.	Audit and verify the aggregation and training process, update the trust of nodes, and feed back the scheduling and constraint information to the local computing layer.

**Table 2:** Description of experimental dataset of HFHE-Cloud model.

Dataset	Data Content Characteristics
Cloud Workload Dataset for Scheduling Analysis, accessed on 6 April 2025 (https://www.kaggle.com/datasets/zoya77/cloud-workload-dataset-for-scheduling-analysis)	It contains the execution records of cloud tasks, and the fields include task ID, CPU, and memory usage, execution duration, task priority, arrival time, and completion status, reflecting the characteristics of cloud scheduling and resource allocation.
Multi-Tenant Cloud Dataset, accessed on 15 March 2025 (https://www.kaggle.com/datasets/ziya07/multi-tenant-cloud-dataset)	It contains multi-tenant virtualization and container operation information, including tenant ID, task ID, CPU and memory allocation ratio, delay, task type, and tenant attributes, reflecting the differences in multi-tenant resource occupation and isolation.
Alibaba Cluster Trace, accessed on 13 December 2018 (v2018) (https://github.com/alibaba/clusterdata)	It contains 8-day job and task logs of 4000 servers, recording information such as scheduling status, task duration, and failure rate, reflecting the real running load of large-scale cloud platforms.
Loghub-A Large Collection of System Log Datasets, accessed on 27 November 2018 (https://github.com/logpai/loghub)	It collects system logs such as HDFS, Spark, Hadoop, and OpenStack, including system calls, error reports, event timestamps, and running status, reflecting the characteristics of multi-system operation and security events.

**Table 3:** Experimental environment.

Type	Item	Configuration
Hardware	CPU	Intel Xeon Gold 6348 × 2, 2.60 GHz, 32 cores
	GPU	NVIDIA A100 40 GB × 2
	RAM	256 GB DDR4
	Storage device	NVMe SSD 2 TB
Software	Operating system	Ubuntu 22.04 LTS (64 bits)
	Programming language	Python 3.10
	Deep learning framework	PyTorch 2.1
	FL frame	Flower 1.8
	HE library	Pyfhel 3.4
	Scheduling and control tool	Kubernetes 1.30

**Table 4:** Homomorphic encryption configuration of HFHE-Cloud and hyperparameter of federated learning training.

Configuration Category	Parameter Term	Setup Description
Homomorphic encryption configuration	Encryption scheme type	Homomorphic encryption based on RLWE
	Clear-text coding mode	Fixed-point encoding
	Modulus scale	q = 2^18^
	Support homomorphic operation	Addition, multiplication, KeySwitch
	Key management mode	Node independent key + aggregate key mapping
Federal learning and training hyperparameter	Number of local training rounds	5
	Learning rate	0.01 (all clients are consistent)
	Batch size	64
	Optimizer	SGD
	Upper limit of global communication rounds	100

**Table 5:** Cross-validation results of equilibrium coefficient of HFHE-Cloud model.

Folds	α	β	γ	λ	Verification Loss	Global Accuracy
First Fold	0.6	0.25	0.1	0.05	0.081	94.30%
Second Fold	0.55	0.3	0.1	0.05	0.078	94.80%
Third Fold	0.5	0.35	0.1	0.05	0.076	95.10%
Fouth Fold	0.55	0.3	0.1	0.05	0.077	94.90%
Fifth Fold	0.5	0.3	0.15	0.05	0.079	94.60%

**Table 6:** HFHE-Cloud verification index system.

Dimension	Index	Explanation
Privacy protection effect	Accuracy	The classification or prediction accuracy of the model on the verification or test set is used to measure the overall learning effect.
	Loss	Measure the error between the predicted value and the real value of the model, and reflect the degree of convergence and optimization of the model.
	Encryption–Decryption Time (s)	The average time consumption of model parameter encryption and decryption in each round of training is used to evaluate the computational efficiency of privacy protection mechanism.
	Proportion of Encryption Overhead	Compared with unencrypted training, the encryption mechanism is used to measure the balance between security and efficiency.
Distributed training performance	Communication Rounds	The number of communication rounds required for the model to achieve stable convergence reflects the training efficiency.
	Upload Latency (s)	The average time taken by the client to upload local encryption parameters to the server reflects the influence of network and node differences.
	Aggregation Time (s)	The average time for a server to complete a homomorphic aggregation operation is used to measure the efficiency of the aggregation algorithm.
	Client Participation Rate	The proportion of nodes participating in uploading in each round of training reflects the parallelism and expansibility of the system.

**Table 7:** Sensitivity analysis results of HFHE-Cloud under different β values.

β Value	Encryption–Decryption Time (s)	Client Participation Rate (%)	Communication Rounds
0.9β	1.56	91.9	47
β	1.59	92.3	46
1.1β	1.66	91.2	48

**Table 8:** Experimental results of HFHE-Cloud expandable ablation.

Client Number	Model Configuration	Accuracy (%)	Encryption–Decryption Time (s)	Upload Latency (s)	Peak Memory Usage (GB)	Communication Rounds
20	HFHE-Cloud-1	94.6	1.61	0.82	6.3	52
20	HFHE-Cloud-2	95.0	2.04	0.79	7.8	47
20	HFHE-Cloud-3	94.4	1.58	0.88	6.1	54
20	HFHE-Cloud	95.1	1.59	0.8	6.5	46
40	HFHE-Cloud-1	94.3	1.91	1.05	6.9	56
40	HFHE-Cloud-2	94.9	2.42	1.01	8.6	50
40	HFHE-Cloud-3	94.1	1.86	1.12	6.8	58
40	HFHE-Cloud	95.0	1.88	1.03	7.1	49
60	HFHE-Cloud-1	94.0	2.20	1.31	7.6	60
60	HFHE-Cloud-2	94.7	2.78	1.26	9.4	53
60	HFHE-Cloud-3	93.8	2.15	1.38	7.5	62
60	HFHE-Cloud	94.9	2.17	1.29	7.9	52

## Data Availability

The data supporting the findings of this study are available within the article.
